# The Impact of Demographics and Comorbidities on Fall Incidence and Prevalence in Older Adults

**DOI:** 10.21203/rs.3.rs-4762014/v1

**Published:** 2024-07-19

**Authors:** Asmaa M Namoos, Nicholas Thomson, Sarah Bradley, Michel Aboutanos

**Affiliations:** Virginia Commonwealth University

**Keywords:** Falls, Older adults, Incidence, Prevalence, Comorbidities

## Abstract

**Introduction:**

Falls among older adults are more than mere accidents; they are a silent epidemic, profoundly impacting the health and well-being of millions of older adults worldwide. This study examines the incidence and prevalence of falls among individuals aged 65 and above, focusing on the influence of demographic factors and comorbid conditions such as hypertension, diabetes mellitus, cancer, and obesity.

**Methods:**

A retrospective cohort study was conducted using data from the TriNetX network at Virginia Commonwealth University Health System (VCUHS) from 2019 to 2023. The study population included 16,400 individuals aged 65 and above who presented with fall-related trauma. Data on demographics, clinical diagnoses, procedures, and comorbid conditions were analyzed using descriptive statistics to evaluate the incidence and prevalence of falls.

**Results:**

The mean age of the study population was 77.3 years, with a higher proportion of females (60.97%) compared to males (39.02%). Despite the larger number of female participants, incidence and prevalence of falls were highest among individuals aged 65–69 years, and fall rates were notably higher among males compared to females. This suggests that while fewer in number, males in our study experienced falls more frequently. Patients with hypertension had the highest incidence proportion (56.67%) and prevalence (75.75%) among comorbid conditions.

**Conclusions:**

Falls among older adults are significantly influenced by demographic factors and comorbid conditions. Hypertension, in particular, is associated with the highest fall risk. These findings highlight the need for targeted interventions to manage comorbidities and reduce fall risks among older adult patients.

## Background

Falls among older adult individuals represent a significant public health concern due to their profound impacts on morbidity, mortality, and quality of life^1^. The World Health Organization reports that falls are the second leading cause of unintentional injury deaths worldwide, particularly affecting adults older than 65 years old^2^. The Centers for Disease Control and Prevention (CDC) identify falls as the leading cause of injury and injury-related deaths in older adults in the United States^3^. Over recent years, the incidence and prevalence of falls have shown a disturbing upward trend^3^. Annually, approximately 36 million older adults experience a fall, leading to over 32,000 fatalities and nearly 3 million emergency department visits^3,4^. This rising trend is influenced by factors including an aging population, prevalent chronic conditions, and inadequate environmental safety^5^.

Research consistently demonstrates that the risk of falls increases with age. Specifically, individuals aged 85 and older experience the highest incidence of falls, with 13.5% reporting fall injuries and approximately 40% falling each year^6,7^. Additionally, gender differences are notable, with women more likely to experience falls than men, potentially due to higher rates of osteoporosis and sarcopenia in women, which contribute to frailty and balance issues^8–10^. In addition to age and gender, racial disparities also play a significant role in the risk of falls among older adults. The studies showed that African American and Hispanic older adult populations have higher rates of fall-related injuries compared to their White counterparts^11–13^. This discrepancy can be attributed to factors such as differences in socioeconomic status, access to healthcare, health care complexity and the prevalence of chronic conditions that exacerbate fall risk^7^.

Moreover, chronic conditions such as hypertension, diabetes mellitus, obesity and cancer are prevalent in this population and are associated with an increased risk of falls^14–17^. A high proportion (75%) of older adult fall patients suffer from hypertension, which can lead to dizziness and balance problems, especially when antihypertensive medications are involved^18^. Diabetes mellitus, affecting about 38% of older adult fall patients, can cause neuropathy and vision impairments, thereby increasing the risk of falls^15,19^. Cancer also presents unique challenges; approximately 43% of older adults fall patients are affected, and treatments such as chemotherapy can lead to muscle weakness, fatigue, and neuropathy, all of which increase susceptibility to falls^20^. Moreover, corticosteroid use, often prescribed for cancer-related conditions, has been linked to adverse fall outcomes, particularly among hypertensive patients, exacerbating fall risks due to muscle wasting and bone loss^21,22^.

Falls among older adults not only pose a significant risk for injury but also underscore a mounting public health challenge indicative of underlying gaps in geriatric health management and preventive care. This retrospective study investigates the incidence and prevalence of falls over the past four years using data extracted from the TriNetX network Virginia Commonwealth University-Health System VCUHS database spanning from 2019 to 2023. With ethical approval secured, the study analyses a cohort of 16,400 individuals aged 65 and above who were presented at hospitals with fall-related trauma. By examining the demographics, including age, gender, ethnicity and racial distribution, alongside comorbidities and risk factors associated with these incidents, this research aims to explore incidence and prevalence of falls related trauma. The findings from this project are critical for developing targeted interventions to reduce the frequency and severity of falls among the older adults. These results will be used to refine fall risk assessments and to shape effective strategies for intervention at both the individual and community levels.

## Method

### Study Design

A retrospective cohort study utilizes data extracted from the TriNetX network VCU database, encompassing 16,400 participants from January 1st, 2019 to December 31st, 2023. The study aims to evaluate the incidence and prevalence of falls among older adults aged 65 years and above who are presented at hospitals with fall-related trauma.

### Data Source

Data for this retrospective cohort study was obtained from the TriNetX network at Virginia Commonwealth University Health System (VCUHS), which aggregates electronic health records (EHRs) exclusively from within the VCUHS network. This database serves as a comprehensive repository of patient information, including demographics such as age, gender, ethnicity and race. These demographic details are used to characterize the study population of older adults aged 65 years and above who experienced fall-related trauma. Clinical diagnoses documented in the EHRs provide insights into prevalent health conditions and comorbidities among the cohort, including hypertension, obesity, diabetes mellitus, and cancer, all of which influence fall risk and outcomes. Procedures recorded in the database illuminate the medical interventions and treatments administered to patients following fall-related injuries, offering context for understanding healthcare management strategies and utilization patterns.

### Data Handling and Analysis

The richness of data within the TriNetX network^23^ facilitated robust statistical analysis. Descriptive statistics were employed to characterize the study population, detailing variables such as age, gender, race, and the prevalence and incidence rate of comorbid conditions. These statistics included means, medians, standard deviations, and frequency distributions. The prevalence and incidence functions on the TriNetX platform were utilized to accurately capture and analyze these metrics, providing a comprehensive overview of the study population’s health status and the impact of comorbid conditions on fall risk. Additionally, patients were identified through ICD-10 codes related to fall injuries, ensuring accurate classification and analysis of fall-related trauma cases.

## Results

### Demographics

The study population consisted of 16,400 individuals aged 65 years and above, with a mean age of 77.3 years (SD ± 8.07), who were presented with fall-related trauma. Gender distribution showed that 60.97% (10,000) of the patients were female, while 39.02% (6,400) were male, with a small fraction (0.06%, or 10 individuals) having an unknown gender. In terms of ethnicity, a vast majority, 96.4% (15,810), were not Hispanic or Latino, 3.04% (500) were Hispanic or Latino, and 0.6% (100) had unknown ethnicity.

Regarding race, 62.92% (10,320) of the patients were White, 31.58% (5,180) were Black or African American, 2.92% (480) were Asian, 0.6% (100) were American Indian or Alaska Native, and 0.18% (30) were Native Hawaiian or Other Pacific Islander. Additionally, 1.89% (310) of the patients identified as another race. This demographic distribution provides a comprehensive overview of the study population, highlighting the diversity and prevalent characteristics within the older adult cohort who experienced falls (Tables 1).

### Incidence and Prevalence by Demographics

Our study examined falls among older adults, analyzing data by age, gender, race, and ethnicity. The highest incidence proportion, 26.78%, and a prevalence of 46.18% were recorded among individuals aged 65–69, with an incidence rate of 2.86 per 1,000 person-days. This trend decreased with age; individuals 85 and older showed the lowest rates: an incidence proportion of 18.06% and prevalence of 33.68%. Males demonstrated higher incidence (26.25%) and prevalence (44.54%) compared to females (22.19% incidence and 42.21% prevalence). Racial analysis revealed the highest incidence among American Indian or Alaska Native individuals at 50%, with a prevalence of 33.33%. Black or African American individuals had an incidence proportion of 24.23% and a prevalence of 47.87%. For ethnicity, Hispanic or Latino individuals had an incidence proportion of 22.22% and a prevalence of 30%, but the highest incidence rate of 4.66 per 1,000 person-days, whereas Non-Hispanic or Latino individuals exhibited an incidence proportion of 23.97% and a prevalence of 43.62%. (Table 2).

### Incidence and Prevalence of Falls among Patients with Diabetes Mellitus Type II

Diabetes Mellitus Type II incidence proportion was 21.21% with a prevalence of 38.33% and an incidence rate of 2.20 cases per 1,000 person-days. Age-specific analysis showed a gradual decrease in incidence proportion from 22.49% in the 65–69 age group to 16.88% in those 85 and older. Similarly, prevalence and incidence rates varied slightly across age groups. Gender analysis revealed that males had a higher incidence proportion (24%) and prevalence (40.44%) compared to females. In terms of racial stratification, American Indian or Alaska Native individuals had the highest rates with an incidence proportion of 50% and prevalence of 66.67%. Ethnicity-wise, Hispanic or Latino individuals exhibited a high incidence rate of 6.56 cases per 1,000 person-days, matching a prevalence of 30%. (Table 3).

### Incidence and Prevalence of Falls among Patients with Hypertension (HTN)

The incidence and prevalence of falls among hypertensive patients, revealing that 56.67% experienced at least one fall, with a prevalence of 75.75% and a high incidence rate of 9.41 per 1,000 person-days. Older age groups, particularly those aged 85 and older, displayed the highest figures: a 65.14% incidence and 80% prevalence. Males showed a greater incidence (62.01%) and prevalence (78.68%) compared to females. Racially, Native Hawaiian or Other Pacific Islander individuals recorded the highest rates, both at 100%, with a notably high incidence rate of 29.33 per 1,000 person-days. Among ethnic groups, Hispanic or Latino individuals reported a 60% incidence and prevalence, demonstrating significant variations across demographics in the impact of hypertension on fall risk. (Table 3)

### Incidence and Prevalence of Falls among Patients with Cancer

The incidence proportion of falls among Patients with Cancer *is* 23.83% and a prevalence of 43.19%, with an incidence rate of 2.63 per 1,000 person-days. Incidence rates were highest among those aged 65–69 years at 26.67% and gradually decreased with age, with the lowest rates observed in individuals 85 and older. Males displayed higher rates compared to females, with a 26.40% incidence and 44.53% prevalence. Racial disparities were pronounced, with Native Hawaiian or Other Pacific Islander individuals showing an incidence and prevalence of 100%, and the highest incidence rate of 10.17 per 1,000 person-days. Hispanic or Latino patients also showed higher rates compared to non-Hispanic or Latino, emphasizing the variability across different ethnic and racial groups (Table 3).

### Incidence and Prevalence of Falls among Patients with High Weight/Obesity

The incidence proportion of high Weight/Obesity is 16.11% with prevalence of 29.88%, with an incidence rate of 1.65 per 1,000 person-days. Incidence proportions were highest in the 65–69 age group at 20.15% and decreased with age, showing the lowest rates in those 85 and older. Females displayed higher incidence proportions (17.01%) and prevalence (31.86%) compared to males. Racial differences were significant, with Native Hawaiian or Other Pacific Islander individuals recording the highest incidence and prevalence at 100%, and an incidence rate of 21.65 per 1,000 person-days. Ethnic variations also showed that Hispanic or Latino individuals had a notably lower incidence proportion and prevalence of 10%, while Non-Hispanic or Latino individuals reported higher figures. These findings underscore the varying impact of obesity on fall risks across different demographic groups (Table 3).

### Summery

The study examined the incidence and prevalence of falls among patients with various comorbidities, including cancer, diabetes mellitus, hypertension (HTN), and high weight/obesity. Among the fall patients, those with hypertension had the highest incidence proportion at 56.67% (0.5667396), with a prevalence of 75.75% (0.75750154) and an incidence rate of 0.94 cases per person-day (0.0009408766). Patients with cancer showed an incidence proportion of 23.71% (0.23707958), a prevalence of 43.11% (0.4311084), and an incidence rate of 0.26 cases per person-day (0.000264396). For those with diabetes mellitus, the incidence proportion was 21.21% (0.21205008), with a prevalence of 38.33% (0.38334355) and an incidence rate of 0.22 cases per person-day (0.000220008). Patients with high weight/obesity exhibited an incidence proportion of 16.11% (0.16105418), a prevalence of 29.88% (0.2988365), and an incidence rate of 0.16 cases per person-day (0.00016495131).

## Discussion

The findings of this study underscore the significant impact of falls among the older adult population, highlighting critical demographic disparities and the prevalence of comorbidities. The data obtained from the TriNetX network at Virginia Commonwealth University Health System (VCUHS) provided a comprehensive overview of the incidence and prevalence of falls among individuals aged 65 and older over a five-year period.

The demographic analysis reveals that falls are most frequent among those aged 65–69, with an incidence of 26.78% and a prevalence of 46.18%. Rates generally decrease with age. Research consistently shows that the risk of falls escalates with age, especially among individuals aged 85 and older, who are notably prone to falling^6^.

Males exhibit higher fall rates (incidence: 26.25%, prevalence: 44.54%) compared to females (incidence: 22.19%, prevalence: 42.21%). Literature indicates that women are more likely to experience falls than men, possibly due to higher rates of osteoporosis and sarcopenia contributing to frailty and balance issues^24(p3) 25,26^.

Among ethnic groups, American Indian or Alaska Native individuals show the highest fall incidence (50%) and rate (5.11 per 1,000 person-days), highlighting significant racial disparities. Hispanic or Latino individuals have an incidence of 22.22% and the highest rate (4.66 per 1,000 person-days), underscoring the need for targeted interventions. Studies reveal that African American and Hispanic older adult populations experience higher rates of fall-related injuries compared to their White counterparts, emphasizing significant disparities in fall risks among ethnic groups^27 12^.

The incidence and prevalence of falls among patients with various comorbidities, including cancer, diabetes mellitus, hypertension (HTN), and high weight/obesity. Our results indicate that among patients who experienced falls, those with hypertension had the highest incidence proportion (56.67%) and prevalence (75.75%), as well as an elevated incidence rate per person-day. Hypertension, in particular, is associated with the highest incidence and prevalence of falls, potentially due to the effects of antihypertensive medications causing dizziness and balance problems^14,18^.

Our study found that cancer patients had an incidence proportion of 23.71%, a prevalence of 43.11%, and an incidence rate of 0.26 fall cases per person-day. These results highlight a significant burden of fall-related incidents among cancer patients.

Similar studies support our findings. Stone et al. reported that falls are common among cancer patients, with a prevalence and incidence rate that closely align with our data^28^. They noted that cancer treatments, increased frailty, and decreased mobility contribute significantly to falling risk among these patients^29^. Additionally, Capone et al. found comparable incidence rates, underscoring the widespread nature of this issue in cancer populations^30^.

The high prevalence rate observed in our study might be due to factors such as cancer treatment side effects, increased frailty, and decreased mobility among patients. The significant incidence proportion indicates a substantial number of new fall cases, underscoring the need for robust prevention strategies.

Our findings, validated by existing literature, emphasize the critical need for ongoing research and effective healthcare policies to reduce fall incidence among cancer patients, ultimately improving their safety and quality of life.

In addition to our findings on cancer patients, our study also revealed significant data for patients with diabetes mellitus. Specifically, the incidence proportion for diabetes patients was 21.21%, with a prevalence of 38.33%, and an incidence rate of 0.22 fall cases per person-day. These findings underscore a substantial burden of fall-related incidents within the diabetic patient population.

Supporting studies show similar trends. Yang et al., conducted a systematic review and found that older adults with diabetes have a significantly higher risk of falls, with an annual incidence of up to 39% in older adults diabetic individuals. This higher incidence is likely due to complications from diabetes such as neuropathy, poor glycemic control, and increased frailty, which impair balance and increase fall risk^31^. Similarly, Tinetti and Speechley observed that the risk of falls is significantly elevated in diabetic individuals, highlighting the need for targeted prevention strategies^32^.

The high prevalence rate in our study may be attributed to the same factors, including poor glycemic control, neuropathy, and other comorbid conditions. These complications can impair balance and mobility, thereby increasing the risk of falls^33^. The significant incidence proportion indicates a substantial number of new fall cases, emphasizing the urgent need for effective fall prevention strategies specifically tailored for diabetic patients.

Also, we examined patients with high weight/obesity, finding an incidence proportion of 16.11%, a prevalence of 29.88%, and an incidence rate of 0.16 cases per person-day. Supporting studies align with our findings. Mitchell et al. reported that obesity increases fall risk due to factors such as decreased mobility and joint instability, reinforcing the need for targeted fall prevention strategies in obese populations^34^.

These findings highlight the significant impact of comorbid conditions on the risk of falls, emphasizing the need for targeted interventions to manage these comorbidities and reduce fall risk among older adult patients.

### Strength

This study has several notable strengths. Utilizing a large and diverse dataset from the TriNetX network at Virginia Commonwealth University Health System (VCUHS) allows for robust analysis with significant sample size, enhancing statistical power and reliability. The use of standardized ICD-10 codes for data extraction ensures consistency and accuracy in identifying diagnoses and comorbidities, minimizing misclassification risk and improving validity. Covering a five-year period, the study provides a longitudinal perspective on the incidence and prevalence of falls among the older adults, allowing for trend observation over time. Additionally, detailed stratification by age, gender, race, and ethnicity identifies high-risk groups, supporting targeted interventions and policies to reduce fall risk and improve outcomes for older adults’ individuals.

### Limitation

This retrospective study faces multiple limitations that may affect the interpretation of its findings. Firstly, the reliance on electronic health records (EHRs) could introduce biases related to incomplete or inaccurate data entries and variations in documentation practices across different providers. Additionally, the use of ICD-10 codes for data extraction might not fully capture the clinical context, possibly leading to misclassification errors. Due to its retrospective design, the study is unable to establish causal relationships.

Another significant limitation is the geographic and institutional restriction of the data to the Virginia Commonwealth University Health System (VCUHS), which may limit the generalizability of the results to other settings. The study also did not account for variables such as the severity of comorbidities and socio-economic factors, which could influence the outcomes, thereby limiting the comprehensiveness of the analysis.

Importantly, the time period of data extraction did not explicitly account for the impact of the COVID-19 pandemic. The years 2020 and 2021 saw a notable decrease in reported falls, likely due to increased reluctance to leave home and the presence of more caregivers at home to assist the elderly. This trend might significantly skew the data, representing a critical limitation for this study period.

## Conclusion

The study underscores the critical impact of comorbid conditions such as hypertension, cancer, diabetes mellitus, and obesity on the incidence and prevalence of falls. Hypertension patients were most affected, exhibiting the highest incidence and prevalence rates. The data reveals a clear correlation between these comorbidities and increased fall risks, necessitating targeted preventive strategies and interventions. By addressing these underlying health issues, healthcare providers may have the opportunity to mitigate fall risks, potentially enhancing patient safety and quality of life. However, it is important to note that our study does not conclusively prove this relationship, and further research is needed to establish direct causal links.

## Figures and Tables

**Table 1:** Demographic Characteristics of the Study Population

Demographic Variable	Category	N	Percentage (%)
**Age Group**	Mean ± SD (77.3 ± 8.07)	16,400	100
**Gender**	Female	10,000	60.97
	Male	6,400	39.02
	Unknown	10	0.06
**Ethnicity**	Not Hispanic or Latino	15,810	96.4
	Hispanic or Latino	500	3.04
	Unknown Ethnicity	100	0.6
**Race**	White	10,320	62.92
	Black or African American	5,180	31.58
	Asian	480	2.92
	American Indian or Alaska Native	100	0.6
	Native Hawaiian or Other Pacific Islander	30	0.18

**Table 2:** Incidence and Prevalence of Falls Stratified by Demographics

Demographic Variable	Category	Incidence Proportion	Prevalence	Incidence Rate (cases/person-day)
**Age Group**	65–69	0.26778242	0.4617737	2.8573073E-4
	70–74	0.2614679	0.4651163	2.7698395E-4
	75–79	0.24022347	0.43514645	2.7270336E-4
	80–84	0.20714286	0.39673913	2.4734615E-4
	85 and older	0.18064517	0.33684212	2.4629655E-4
**Gender**	Female	0.22192152	0.42211056	2.3936154E-4
	Male	0.2625	0.44541408	3.0896158E-4
	Unknown Gender	1.0	1.0	0.011947432
**Race**	American Indian or Alaska Native	0.5	0.33333334	5.108818E-4
	Asian	0.2857143	0.4	3.583138E-4
	Black or African American	0.24225353	0.47868216	2.4403581E-4
	Native Hawaiian or Other Pacific Islander	1.0	1.0	0.01017294
	Unknown Race	0.25	0.29032257	3.816898E-4
	White	0.23954372	0.4163424	2.7602146E-4
	Other Race	0.175	0.33333334	2.3377126E-4
**Ethnicity**	Hispanic or Latino	0.22222222	0.3	4.6598323E-4
	Not Hispanic or Latino	0.23972602	0.4361905	2.6624283E-4
	Unknown Ethnicity	0.1904762	0.30612245	2.1566889E-4

**Table 3:** Comorbidities Stratified Results by Demographics

Diabetes Mellites type DM II				
Demographic Variable	Category	Incidence Proportion	Prevalence	Incidence Rate (cases/person-day)
**Overall**	2019-01-01–2023-12-31	0.21205008	0.38334355	2.2008002E-4
	65–69	0.2248996	0.40978593	2.2014206E-4
	70–74	0.23043478	0.41196012	2.2894268E-4
	75–79	0.22872343	0.39330545	2.4665735E-4
	80–84	0.21333334	0.35869566	2.3355192E-4
	85 and older	0.16875	0.3	2.1087358E-4
**Gender**	Female	0.19512194	0.36984923	1.9574359E-4
	Male	0.24	0.40438873	2.6387515E-4
	Unknown Gender	1.0	1.0	0.011947432
**Race**	American Indian or Alaska Native	0.5	0.66666667	7.041573E-4
	Asian	0.25	0.3	2.7848172E-4
	Black or African American	0.2764706	0.5232558	2.7974666E-4
	Native Hawaiian or Other Pacific Islander	1.0	1.0	0.010050251
	Unknown Race	0.2413793	0.29032257	3.7190918E-4
	White	0.1863426	0.3151751	1.9083255E-4
	Other Race	0.24324325	0.41666666	3.7255973E-4
**Ethnicity**	Hispanic or Latino	0.3	0.3	6.556804E-4
	Not Hispanic or Latino	0.21026894	0.384127	2.164523E-4
	Unknown Ethnicity	0.26190478	0.3877551	3.1567106E-4
Hypertension (HTN)				
Demographic Variable	Category	Incidence Proportion	Prevalence	Incidence Rate (cases/person-day)
**Overall**		0.5667396	0.75750154	9.408766E-4
	65–69	0.5277778	0.74006116	7.978435E-4
	70–74	0.5903614	0.77408636	9.3713007E-4
	75–79	0.6	0.7824268	0.0010945909
	80–84	0.64761907	0.79891306	0.0012591217
	85 and older	0.6513761	0.8	0.0016007035
**Gender**	Female	0.5332136	0.7386935	8.3775294E-4
	Male	0.6201117	0.7868339	0.0011324827
	Unknown Gender	1.0	1.0	0.90909094
**Race**	American Indian or Alaska Native	0.5	0.6666667	8.45666E-4
	Asian	0.5	0.6	8.378484E-4
	Black or African American	0.6492891	0.85658914	0.0011636964
	Native Hawaiian or Other Pacific Islander	1.0	1.0	0.029325513
	Unknown Race	0.5769231	0.6451613	0.0011215121
	White	0.5470219	0.71789885	8.729749E-4
	Other Race	0.45454547	0.625	0.0010088985
**Ethnicity**	Hispanic or Latino	0.6	0.6	0.0015579155
	Not Hispanic or Latino	0.5650173	0.76	9.361068E-4
	Unknown Ethnicity	0.5945946	0.71428573	9.916029E-4
Cancer				
Demographic Variable	Category	Incidence Proportion	Prevalence	Incidence Rate (cases/person-day)
Overall		0.23832923	0.4318876	2.634043E-4
Age Group				
	65–69	0.26666668	0.46341464	2.812224E-4
	70–74	0.2614679	0.46357617	2.7299204E-4
	75–79	0.24022347	0.43333334	2.704202E-4
	80–84	0.20567375	0.39673913	2.451714E-4
	85 and older	0.18064517	0.33684212	2.447009E-4
Gender				
	Female	0.22297297	0.4232698	2.38103E-4
	Male	0.26403326	0.4453125	3.0847368E-4
	Unknown Gender	1.0	1.0	0.011947432
Race				
	White	0.24050634	0.41650486	2.7436882E-4
	Black or African American	0.24507043	0.48069498	2.4444156E-4
	Asian	0.2857143	0.4	3.5910512E-4
	Native Hawaiian or Other Pacific Islander	1.0	1.0	0.01017294
	American Indian or Alaska Native	0.5	0.33333334	5.108818E-4
	Unknown Race	0.25	0.29032257	3.751199E-4
	Other Race	0.175	0.33333334	2.3280564E-4
Ethnicity				
	Hispanic or Latino	0.22222222	0.3	4.4066453E-4
	Not Hispanic or Latino	0.24102564	0.43726236	2.6529646E-4
	Unknown Ethnicity	0.1904762	0.3	2.1467751E-4
High Weight/Obesity				
Demographic Variable	Category	Incidence Proportion	Prevalence	Incidence Rate (cases/person-day)
**Overall**		0.16105418	0.2988365	1.6495131E-4
	65–69	0.20152092	0.35779816	2.0036096E-4
	70–74	0.18032786	0.33554816	1.773495E-4
	75–79	0.15686275	0.28033474	1.6027583E-4
	80–84	0.09580839	0.18478261	9.729123E-5
	85 and older	0.06818182	0.1368421	7.991337E-5
**Gender**	Female	0.17013463	0.31859297	1.722493E-4
	Male	0.14754099	0.26802507	1.5379589E-4
	Unknown Gender	0.0	0.0	0.0
**Race**	American Indian or Alaska Native	0.5	0.6666667	6.4114894E-4
	Asian	0.11111111	0.2	1.1108396E-4
	Black or African American	0.18734178	0.37790698	1.8117481E-4
	Native Hawaiian or Other Pacific Islander	1.0	1.0	0.021645023
	White	0.15410574	0.26750973	1.594208E-4
	Unknown Race	0.13333334	0.16129032	1.8771234E-4
	Other Race	0.11904762	0.22916667	1.4704412E-4
**Ethnicity**	Hispanic or Latino	0.1	0.1	1.9410692E-4
	Not Hispanic or Latino	0.16259542	0.30349207	1.657288E-4
	Unknown Ethnicity	0.1521739	0.20408164	1.6150318E-4

## Data Availability

The datasets generated and/or analyzed during the current study were extracted from the TriNetX database and are available upon request from the corresponding author due to privacy and ethical restrictions.

## References

[1] Vaishya R, Vaish A. Falls in Older Adults are Serious. Indian J Orthop. 2020;54(1):69–74. doi:10.1007/s43465-019-00037-x 32257019 PMC7093636

[2] Falls. Accessed June 30, 2024. https://www.who.int/news-room/fact-sheets/detail/falls

[3] CDC. About Older Adult Fall Prevention. Older Adult Fall Prevention. Published June 13, 2024. Accessed June 30, 2024. https://www.cdc.gov/falls/about/index.html

[4] Falls in Older Adults - Geriatrics. Merck Manual Professional Edition. Accessed June 30, 2024. https://www.merckmanuals.com/professional/geriatrics/falls-in-older-adults/falls-in-older-adults

[5] Appeadu MK, Bordoni B. Falls and Fall Prevention in Older Adults. In: StatPearls. StatPearls Publishing; 2024. Accessed June 30, 2024. http://www.ncbi.nlm.nih.gov/books/NBK560761/32809596

[6] Prevention I of M (US) D of HP and D, Berg RL, Cassells JS. Falls in Older Persons: Risk Factors and Prevention. In: The Second Fifty Years: Promoting Health and Preventing Disability. National Academies Press (US); 1992. Accessed July 1, 2024. https://www.ncbi.nlm.nih.gov/books/NBK235613/25144081

[7] Qin Z, Baccaglini L. Distribution, Determinants, and Prevention of Falls Among the Elderly in the 2011–2012 California Health Interview Survey. Public Health Rep. 2016;131(2):331–339. 26957668 10.1177/003335491613100217 PMC4765982

[8] Lee DY, Shin S. Association of Sarcopenia with Osteopenia and Osteoporosis in Community-Dwelling Older Korean Adults: A Cross-Sectional Study. J Clin Med. 2021;11(1):129. doi:10.3390/jcm11010129 35011870 PMC8745168

[9] Stevens J, Sogolow E. Gender differences for non-fatal unintentional fall related injuries among older adults. Inj Prev. 2005;11(2):115–119. doi:10.1136/ip.2004.005835 15805442 PMC1730193

[10] Risk Factors for Falls Among Seniors: Implications of Gender | Request PDF. ResearchGate. doi:10.1093/aje/kwu268 25700887

[11] Bergen G. Falls and Fall Injuries Among Adults Aged ≥65 Years — United States, 2014. MMWR Morb Mortal Wkly Rep. 2016;65. doi:10.15585/mmwr.mm6537a2 27656914

[12] Nicklett EJ, Taylor RJ. Racial/ethnic predictors of falls among older adults: The Health and Retirement Study. J Aging Health. 2014;26(6):1060–1075. doi:10.1177/0898264314541698 25005171 PMC4227632

[13] Falls in African American and White Community-Dwelling Elderly Residents | Request PDF. Accessed June 30, 2024. https://www.researchgate.net/publication/11290209_Falls_in_African_American_and_White_Community-Dwelling_Elderly_Residents 10.1093/gerona/57.7.m473 12084812

[14] Abu Bakar AAZ, Abdul Kadir A, Idris NS, Mohd Nawi SN. Older Adults with Hypertension: Prevalence of Falls and Their Associated Factors. Int J Environ Res Public Health. 2021;18(16):8257. doi:10.3390/ijerph18168257 34444005 PMC8392439

[15] Risk factors for falls in older adults with diabetes mellitus: systematic review and meta-analysis | BMC Geriatrics | Full Text. Accessed June 30, 2024. 10.1186/s12877-024-04668-0 PMC10900672 38413865

[16] G R Neri S, S Oliveira J, B Dario A, M Lima R, Tiedemann A. Does Obesity Increase the Risk and Severity of Falls in People Aged 60 Years and Older? A Systematic Review and Meta-analysis of Observational Studies. J Gerontol A Biol Sci Med Sci. 2020;75(5):952–960. doi:10.1093/gerona/glz272 31750880

[17] Balance Problems and Falls | Cancer-related Side Effects | American Cancer Society. Accessed June 30, 2024. https://www.cancer.org/cancer/managing-cancer/side-effects/falls.html

[18] Tinetti ME, Han L, Lee DSH, Antihypertensive Medications and Serious Fall Injuries in a Nationally Representative Sample of Older Adults. JAMA Intern Med. 2014;174(4):588–595. doi:10.1001/jamainternmed.2013.14764 24567036 PMC4136657

[19] Milanesi A, Weinreb JE. Diabetes in the Elderly. In: Feingold KR, Anawalt B, Blackman MR, , eds. Endotext. MDText.com, Inc.; 2000. Accessed June 30, 2024. http://www.ncbi.nlm.nih.gov/books/NBK279147/

[20] Morris R, Lewis A. Falls and Cancer. Clin Oncol. 2020;32(9):569–578. doi:10.1016/j.clon.2020.03.011 32291190

[21] Jung YS, Suh D, Kim E, Park HD, Suh DC, Jung SY. Medications influencing the risk of fall-related injuries in older adults: case–control and case-crossover design studies. BMC Geriatr. 2023;23(1):452. doi:10.1186/s12877-023-04138-z 37481554 PMC10363319

[22] van Staa TP, Leufkens HGM, Abenhaim L, Zhang B, Cooper C. Oral corticosteroids and fracture risk: relationship to daily and cumulative doses. Rheumatology. 2000;39(12):1383–1389. doi:10.1093/rheumatology/39.12.1383 11136882

[23] Real-world data for the life sciences and healthcare | TriNetX. Accessed July 1, 2024. https://trinetx.com/?utm_id=130&utm_campaign=enterprise&utm_source=emagine&utm_medium=paid-display&utm_content=evergreen&gad_source=1&gclid=CjwKCAjwp4m0BhBAEiwAsdc4aAuhCMohbUTSdxCaY02DQr-dYQlT2zUMo-vLvtAwBua2dG1F5XDPyxoC_0cQAvD_BwE

[24] Greco EA, Pietschmann P, Migliaccio S. Osteoporosis and Sarcopenia Increase Frailty Syndrome in the Elderly. Front Endocrinol. 2019;10:255. doi:10.3389/fendo.2019.00255 PMC6491670 31068903

[25] Martin FC, Ranhoff AH. Frailty and Sarcopenia. In: Falaschi P, Marsh D, eds. Orthogeriatrics: The Management of Older Patients with Fragility Fractures. 2nd ed. Springer; 2021. Accessed July 1, 2024. http://www.ncbi.nlm.nih.gov/books/NBK565582/33347100

[26] Asavamongkolkul A, Adulkasem N, Chotiyarnwong P, Prevalence of osteoporosis, sarcopenia, and high falls risk in healthy community-dwelling Thai older adults: a nationwide cross-sectional study. JBMR Plus. 2024;8(2):ziad020. doi:10.1093/jbmrpl/ziad020 38505534 PMC10945715

[27] Wehner-Hewson N, Watts P, Buscombe R, Bourne N, Hewson D. Racial and Ethnic Differences in Falls Among Older Adults: a Systematic Review and Meta-analysis. J Racial Ethn Health Disparities. 2022;9(6):2427. doi:10.1007/s40615-021-01179-1 34786654 PMC9633486

[28] Stone CA, Lawlor PG, Savva GM, Bennett K, Kenny RA. Prospective study of falls and risk factors for falls in adults with advanced cancer. J Clin Oncol Off J Am Soc Clin Oncol. 2012;30(17):2128–2133. doi:10.1200/JCO.2011.40.7791 22585687

[29] Kenis C, Decoster L, Flamaing J, Incidence of falls and fall-related injuries and their predictive factors in frail older persons with cancer: a multicenter study. BMC Geriatr. 2022;22(1):877. doi:10.1186/s12877-022-03574-7 36402961 PMC9675153

[30] Campbell G, Wolfe RA, Klem ML. Risk factors for falls in adult cancer survivors: An integrative review. Rehabil Nurs Off J Assoc Rehabil Nurses. 2018;43(4):201–213. doi:10.1097/rnj.0000000000000173 PMC6029723 29957697

[31] Yang Y, Hu X, Zhang Q, Zou R. Diabetes mellitus and risk of falls in older adults: a systematic review and meta-analysis. Age Ageing. 2016;45(6):761–767. doi:10.1093/ageing/afw140 27515679

[32] Tilling LM, Darawil K, Britton M. Falls as a complication of diabetes mellitus in older people. J Diabetes Complications. 2006;20(3):158. 16632235 10.1016/j.jdiacomp.2005.06.004

[33] Freire LB, Brasil-Neto JP, da Silva ML, Risk factors for falls in older adults with diabetes mellitus: systematic review and meta-analysis. BMC Geriatr. 2024;24(1):201. doi:10.1186/s12877-024-04668-0 38413865 PMC10900672

[34] Mitchell RJ, Lord SR, Harvey LA, Close JCT. Obesity and falls in older people: mediating effects of disease, sedentary behavior, mood, pain and medication use. Arch Gerontol Geriatr. 2015;60(1):52–58. doi:10.1016/j.archger.2014.09.006 25307955

